# Georgian Grapes and Wines as a Source of Phenolic Compounds: Composition, Antioxidant Activity, and Traditional Winemaking †

**DOI:** 10.3390/molecules31020303

**Published:** 2026-01-15

**Authors:** Valentina Mittova, Zurab R. Tsetskhladze, Nino Motsonelidze, Rosanna Palumbo, Giovanni N. Roviello

**Affiliations:** 1 Scientific-Research Institute of Experimental and Clinical Medicine, University Geomedi, 4 King Solomon II Str., Tbilisi 0114, Georgia; 2 Faculty of Medicine, University Geomedi, 4 King Solomon II Str., Tbilisi 0114, Georgia; 3 Institute of Biostructures and Bioimaging (IBB), Italian National Council for Research (CNR), Area di Ricerca Site and Headquarters, Via Pietro Castellino 111, 80131 Naples, Italy

**Keywords:** Georgia, wine, phenolic compounds, Rkatsiteli, Saperavi

## Abstract

Georgia is recognized as one of the world’s earliest known centers of grape cultivation and wine production, as well as the home of 525 indigenous grape varieties. Phenolic compounds are a diverse group of secondary metabolites which are present in both grapes and wine, with the phenolic derivatives determining the organoleptic properties and the antioxidant activity of the resulting wines. Remarkably, the content and composition of phenolic compounds in wine are mainly influenced by the grape variety and the winemaking method. In this context, herein we review the present knowledge on the phenolic composition of the most common Georgian grape varieties and discuss available molecular insights on the resulting wines. The comparison of traditional European and traditional Georgian “qvevri” winemaking methods revealed that this method provides high antioxidant activity of Georgian wines, as well as a unique phenolic composition of red and white Georgian wines.

## 1. Introduction

The antioxidant properties of natural products have garnered significant attention in recent years, since antioxidants have various pharmacological properties such as cardioprotective, neuroprotective, anti-inflammatory, anticancer, and anti-aging activities [1,2]. In this context, grapes are a rich source of antioxidants [3], and the beneficial effect of these compounds has been related, at least in part, to the “French Paradox”, i.e., the low incidence of coronary heart disease in the population of the Mediterranean region which was attributed to the regular consumption of red wines [4]. The “French Paradox” was linked to the high content of phenolic compounds, mainly flavonoids, in red wine, which are endowed with diverse health-protective properties and represent some of the most potent nutraceuticals in food and phytopharmaceutical products [5,6]. Importantly, the total content and composition of wine phenolic compounds is affected by the winemaking process, grape cultivar, conditions during maturation and storage [7]. In this frame, Georgia and, more broadly, the Caucasus region, are considered the cradle of the world’s winemaking, the primary center of domestication of the grape [8], with the Georgian grape gene pool including more than 500 varieties [9]. Interestingly, the Georgian traditional “qvevri” method used for wine production contributes to a significantly higher antioxidant capacity compared to wines produced by the European method [7,10,11]. However, the information about the phytochemical profile of Georgian wines is quite limited in the scientific literature. Accordingly, the objective of this study was to perform a comparative analysis of the available data on the phenolic composition of Georgian grape varieties and wines produced by both European and “qvevri” methods. The information presented in this review is primarily based on studies where wines produced by the European and “qvevri” methods were investigated in the same research, using the same methods, which, in our opinion, provides a justified background for comparison. This review provides a structured overview of the phenolic composition of Georgian grape varieties and wines, with particular attention to their antioxidant potential and the influence of traditional winemaking practices.

### 1.1. Methodology

As for the methodology adopted, the narrative literature review was conducted according to the methodology proposed in [12]. At the first stage, a literature search was carried out in PubMed, Scopus, Web of Science, and Google Scholar databases. These sources were chosen due to their extensive coverage of high-quality, peer-reviewed journals. The search was performed in English, Georgian, and Russian, using the following keywords: 1. “Antioxidant activity”; 2. “Georgian wine”; 3. “Phenolic compounds”, 4. “Flavonoids”, 5. “Phenolic acids” and their combination, incorporating Boolean operators (OR, AND). The initial search provided 1023 articles. To ensure data quality, following the initial search, retracted publications and duplicate records were excluded from the literature analysis (29). During the next stage, screening of the titles of selected articles and exclusion of inappropriate articles was performed (451), followed by screening of the remaining articles based on the abstract. At this stage, another 189 articles were excluded. The final selection of articles was performed based on the full text, and 138 articles were included in the review. Remarkably, more than 30% of the cited references originate from publications in 2020 or later, testifying to the strong and continuing scientific interest in the theme discussed in this review.

### 1.2. Grape Origin and the First Domestication Center

Grape is a special crop because of its long-standing historical ties to the evolution of human civilization. The primary product, wine, has traditionally played a significant role in Mediterranean people’s lives and was regarded as a drink of the gods [8]. The genus *Vitis* L. (grape), one of the 16 genera in the family *Vitaceae*, includes 68 species [13]. The same genus is present in 10 distribution areas, all in the northern hemisphere. Moreover, in detail, 29 species have been described in North America in 5 distribution areas, and 11 species were demonstrated for 4 distribution areas in Asia. Only one, *Vitis vinifera*, is distributed over a wide range, including the Mediterranean, sub-Mediterranean, and Caucasian floristic regions with a spread toward the Pontic, Caspian, and Central Asiatic areas [14] and is suggested to have first appeared around 65 million years ago [15]. The *V. vinifera* species includes both cultivated (*V. vinifera* subsp. *sativa*) and wild (*V. vinifera* subsp. *sylvestris*) subspecies, with the wild subspecies being considered the progenitor of the subspecies *sativa*, domesticated since its fermented juice, or wine, is superior to other fleshy fruits in terms of both alcohol content and flavor [16]. For a long time, the domestication of the grapevine from *V. vinifera* subsp. *sylvestris* populations were believed to have occurred 6000–8000 years ago in the Transcaucasian region [17,18]. However, more recently, a theory of a four-stage domestication process was proposed, suggesting that the domestication started much earlier, around 20,000 years ago, when South Caucasian human populations started to manage and harvest the local *V. vinifera* subsp. *sylvestris* population [19]. From the primary center of domestication, cultivated varieties were distributed throughout Europe and Mediterranean areas, following the spread of civilizations [20]. This pattern of domestication has been confirmed by molecular data [21]; however, secondary domestication events, involving both local wild grapevines and varieties introduced from the primary center, also undoubtedly occurred at various locations [22]. A Russian botanist, Nikolai Vavilov, was the first to claim that the earliest “wine culture” in the world emerged in Transcaucasia, an area comprising modern Georgia, Armenia, and Azerbaijan [23].

### 1.3. Grapevine Cultivation in Georgia

**Figure 1:**
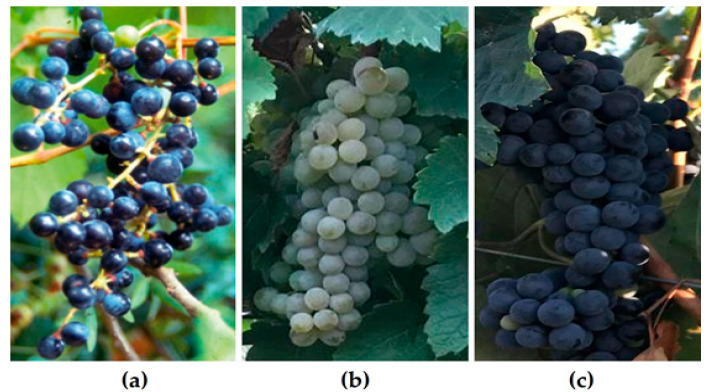
(**a**) Wild grape *Vitis vinifera* subsp. *sylvestris*; (**b**) Rkatsiteli and (**c**) Saperavi grape varieties, Farna’s Wine Cellar, the village of Kistauri, Georgia (42.0126690, 45.2817010).

Georgia is a country characterized by mountainous terrain in the southwestern region of the Caucasus. The Greater Caucasus mountain range forms its northern border, while the Lesser Caucasus defines its southern boundary. The country faces the Black Sea in the west and the Caspian depression in the east. The location at the turn of the Mediterranean, the arid Aral-Caspian depression and the Forward Asia highlands, coupled with a continental climate, have resulted in diverse topography, varied landscapes, and a wide range of animal and plant life [24]. The Greater Caucasus mountains shield Georgia from the direct intrusion of cold air masses originating from the north, whereas the Black Sea moderates temperature variations and affects precipitation levels, particularly in western Georgia [25]. According to the solar radiation regimes, Georgia is in the subtropical belt. Based on the characteristics of atmospheric circulation and associated meteorological conditions, the territory of Georgia is separated into two regions and one sub-region: (1) subtropical marine–humid Western Georgia; (2) a transitional zone from sub-tropical continental to marine climate, with the latter including a transitional sub-region ranging from dry subtropical to moderate-humid climate, which is Eastern Georgia [25,26]. The central steppe part of Georgia’s southern highlands is a subzone with a more continental climate and less atmospheric precipitation than the surrounding regions [27]. Owing to these geographical features, different climatic conditions are characteristic of the wine-growing regions of Georgia, ranging from hot summer continental climates to warm summer continental and hemiboreal climates [27]. Climatic conditions substantially affect grape development, cell wall composition and phenolic composition [28]. The oldest biomolecular archaeological evidence for grape wine and viniculture in the South Caucasus region dates to the early Neolithic period (ca. 6000–5000 BC), according to chemical analyses of organic compounds absorbed into pottery from diverse Georgian archeological sites [20]. Nowadays, Georgia counts 48,000 hectares of vineyards, and the production of wine and table grapes amounts to 159,000 and 8000 tons, respectively [27]. The country is home to 525 autochthonous white, black, red, pink and gray grape varieties [29] as well as many populations of wild *Vitis vinifera* ssp. *sylvestris* grapes [4]. The top white grape variety is Rkatsiteli (19,741 ha), cultivated in Kakheti, and the top red grape variety is Saperavi (3704 ha) (Figure 1), cultivated in Kakheti and Eastern Georgia [9].

The soils where the main commercial vineyards grow include several varieties of cinnamonic soils, such as meadow cinnamonic, gray cinnamonic, and cinnamonic forest soils. In this respect, it was shown that commercial *Vitis vinifera* L. subsp. *sylvestris* (C.C. Gmel.) Hegi varieties and wild grapevine individuals favor deep, fertile, and evolved soils, mainly alluvial and cinnamonic types [30]. Hence, famous Georgian wines like Tsinandali, Vazisubani, Akhasheni, Gurjaani, Manavi and Kardanakhi originate from grape varieties grown on cinnamonic soils [26]. In Georgia, three traditional training methods are used for the cultivation of grapevines and their varieties. The so-called “babilo” is the method employed for old grapevines, with stems more than 20 cm in diameter, which clamber on trees, native to the Guria region. The “maghlari” method makes use of vines that climb tree trunks (alder, persimmon, mulberry, cherry, beech, chestnuts, etc.) distributed mainly in humid areas of western and southern Georgia, protecting grape varieties from fungal diseases. Finally, the “dablari” method creates typical vineyards found in commercial areas, grapevines grown close to the ground, without the use of a tree for support, with this method being used mainly in western and eastern Georgia [31].

## 2. Polyphenolic Compounds in Georgian Grapes

Plant polyphenolic compounds comprise a large group of more than 8000 compounds, with the phenolic hydroxyl groups being the common structural feature [32]. Polyphenolic compounds are one of the secondary metabolites widely distributed in plants [33]. They are derivatives of the pentose phosphate, shikimate, and phenylpropanoid pathways.

**Figure 2:**
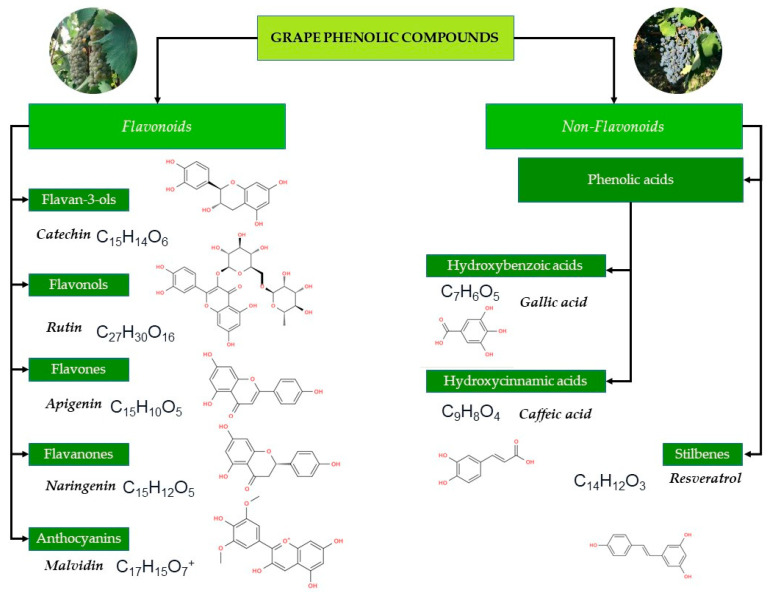
Classification of grape phenolic compounds. The diagram illustrates the major classes of phenolics found in *Vitis vinifera* grapes and wines, including flavonoids and non-flavonoid compounds.

Due to the presence of hydroxyl and carboxyl groups in their molecules, polyphenolic compounds conjugate sugars, organic acids, and amines, forming glycosidic, methylated, methoxylated, and acylated compounds of phenolic nature [34]. Polyphenolic compounds can be divided into two major groups: flavonoids and non-flavonoids [35] (Figure 2). The core structure of flavonoids is a 2-phenylbenzopyranone, in which the three-carbon bridge between the phenyl groups is commonly cyclized with oxygen [36]. The major classes of flavonoids are distinguished based on the degree of unsaturation and the degree of oxidation of the three-carbon segment [37].

The classes of flavonoids revealed in grapes include flavan-3-ols, flavonols, flavones, flavanones, and anthocyanins [35]. The group of non-flavonoid phenolic compounds comprises phenolic acids, subdivided into derivatives of hydroxybenzoic (gallic acid, vanillic, syringic acids) and hydroxycinnamic acids (ferulic, caffeic, coumaric acids) and stilbenes (resveratrol) [38]. Polyphenolic compounds in grapes are mainly found in the skin (28–35%), seeds (60–70%), and stems, while their content in the pulp is much lower and does not exceed 10% [39]. While grape skins contain mainly flavan-3-ols, flavonols and anthocyanins [40]. The stalk contains primarily stilbenes and flavanols [41] and seeds present a considerable amount of flavan-3-ols, flavanols and phenolic acids [42]. Remarkably, anthocyanins are polyphenols more representative of red grapes, while flavonols are more characteristic of white grapes [43].

### 2.1. Flavonoids

Flavonoids compose a large family of secondary plant metabolites with around 6000 compounds [44]. The diversity in their chemical structures contributes to their broad range of physiological and biological activities. The most common flavonoids, identified in grapes worldwide, are flavan-3-ols (catechin, epicatechin, epicatechin-3-*O*-gallate), flavonols (3-*O*-glycosides of quercetin, kaempferol, myricetin, laricitrin, isorhamnetin and syringetin), dihydroflavonols, and anthocyanins (3-*O*-monoglucosides or 3,5-*O*-diglucosides of malvidin, cyanidin, peonidin, delphinidin, and pelargonidin) [4]. Flavonoids are present primarily in the skin, and their synthesis begins during the flowering stage, decreasing during fruit development and resuming during fruit ripening [45]. Flavan-3-ols are located in both the grape skin and the seed [46] and are characterized by a 2-phenyl-3,4-dihydro-2H-chromene skeleton that is hydroxylated at position 3 of ring C, typically bearing additional hydroxyl substitutions on the A and B aromatic rings, which confer strong hydrogen-bonding capacity and redox activity [47]. They have two chiral centers at positions 2 and 3, creating chiral diastereomers with four possible configurations: two enantiomers for each epimer [48]. Flavan-3-ols are present in grapes mainly as catechin and epicatechin [49]. Interestingly, in grape seeds, the most abundant flavan-3-ols are catechin, epicatechin, and epicatechin 3-*O*-gallate [50]. While flavan-3-ols constitute up to 56% of the total phenolics found in white grape varieties, in red grapes their content is only 13–30% [51]. In Georgian grape varieties, the most common flavan-3-ols were confirmed to be catechin and epicatechin, identified in the skin, seeds, and stalk of the white grape variety Tsolikauri, where the highest abundance of both compounds was revealed in the seeds (catechin, 3.131 mg/g dry weight and epicatechin, 0.211 mg/g dry weight) [52]. Gallocatechin, epicatechin, catechin, and epicatechin gallate were also identified in the skin and stalk of the white grape variety Rkatsiteli, while seeds of the same variety contained epicatechin, catechin, and epicatechin gallate [53]. Catechin was the most abundant flavanol in the skin, stalk and seeds of the same white grape variety [53]. On the other hand, catechin hydrate was revealed in the skin and seeds of the red grape variety Saperavi, with its highest abundance being demonstrated in the skin (1200 mg/kg) compared with the seeds [54], Table 1.

Flavonols are the second most abundant flavonoids in grapes, characterized by a 3-hydroxyflavone backbone, present only as 3-*O*-glycosides in grape skins, but can also be found as aglycones (quercetin, kaempferol) in extracts as a result of acid hydrolysis during processing and storage, which alters their solubility and biological activity [59]. In grapes, kaempferol, quercetin, myricetin, and their methylated forms, isorhamnetin, laricitrin, and syringetin were also revealed [60]. In fact, both red and white grapes contain the already mentioned quercetin, kaempferol, and isorhamnetin derivatives, whereas myricetin derivatives are present only in red varieties [61]. In the Georgian white grape variety Tsolikauri, among the different flavon-3-ols, rutin was identified in the stalk and skin, where it was most abundant (0.149 mg/g) [52]. Quercetin was present in the stalks and seeds of the same variety, and its content in the seeds was the highest (0.102 mg/g) [52]. Structurally, flavones present a 4H-chromen-4-one moiety with a phenyl substituent at position 2. Among this class of flavonoids, the most studied compounds are luteolin and apigenin [62], both considered minor flavonoids in grapes. Luteolin accumulation has been demonstrated in the skin of the European red grape variety Merlot [63]; however, flavones were not discovered in Georgian berries. The flavanone structure is characterized by a benzopyranone core substituted at the C2 position, with possible substitution on the aryl backbone of the benzopyranone core [64]. They are generally glycosylated at the 7 position by a glucoside or disaccharide [65]. The following eight flavanones have been shown in *Vitis vinifera*: taxifolin, taxifolin-*O*-pentoside, taxifolin-3-*O*-glucoside, taxifolin-3-*O*-rhamnoside, hesperetin, eriodictyol-7-*O*-glucoside, naringenin, and naringenin-7-*O*-glucoside [66]. Anthocyanins have the basic structure of the flavylium ion (2-phenyl-benzopyrylium chromophore) [67], a semi-planar framework with a dihedral angle (<10°) between the B ring and the benzopyrylium [68]. Usually, anthocyanidin glycosides are 3-monoglycosides and 3,5-diglycosides in which sugar moieties are attached to the hydroxyl groups, further modulating their solubility and color expression [69]. Anthocyanins mainly accumulate in the berry skin, and they are the group of flavonoids most involved in berry pigmentation (i.e., berries’ skin; red, purple, and blue colors) [70], but in some varieties known as “teinturier” (or dyed), anthocyanin pigments are found to accumulate in the pulp [71]. The *Vitis vinifera* L. cultivars are usually characterized by the presence of 3-mono-glucoside, 3-acetyl-glucoside, and 3-*p*-coumaryl-glucoside [72] and derivatives of the aglycones delphinidin, peonidin, petunidin, cyanidin, and malvidin [73]. Remarkably, in the skin of the Georgian red grape variety Saperavi, delphinidin, petunidin, malvidin, peonidin, and delphinidin-3-*O*-glucoside were revealed, with the most abundant compounds being malvidin (34.2% of the total anthocyanin content in the grape skin) and petunidin (19.4% of the total anthocyanin content in the grape skin) [56]. On the other hand, in the pulp of the Georgian red grape variety Chkhaveri, the dominant anthocyanins were malvidin-3-*O*-glucoside, peonidin-3-*O*-glucoside, cyanidin-3-*O*-glucoside, petunidin-3-*O*-glucoside, delphinidin-3-*O*-glucoside, and flavon-3-ols such as catechin were also identified [74].

### 2.2. Non-Flavonoids

Most phenolic acids of grapes are hydroxycinnamic acids and hydroxybenzoic acids. Hydroxycinnamic acids are phenylpropanoids whose skeleton consists of a benzene ring with a 3-carbon aliphatic chain, with one or more hydroxyl groups often esterified to the aliphatic alcohol ester, which modulates their radical-scavenging capacity and metal-chelating properties [75]. They include *p*-coumaric acid, caffeic acid, ferulic acid, and sinapic acid. Hydroxybenzoic acids are characterized by a C6-C1 carbon skeleton composed of a benzene ring on which an aliphatic chain is attached to a carbon, and they include vanillic, syringic, gentisic, and gallic acids, whose hydroxylation and methoxylation patterns modulate their biological activities [75]. The main phenolic acids in grapes comprise the derivatives of hydroxycinnamic acids, as well as *p*-coumaric, caffeic, and ferulic acids [76]. They are typically present as esters with tartaric acid. Ferulic and *p*-coumaric acids bind to cell wall polysaccharides, and these bonds function as cross-links between pentose chains [57]. The study of phenolic compounds in the grape bunch (stem, grape skin, seeds) of the Georgian autochthonous vine variety Tsolikauri indicated the highest content of hydroxycinnamic acids, caffeic and ferulic in the bunch stem (0.026 mg/g) and seeds, respectively (0.02 mg/g). The highest content of hydroxybenzoic acids was observed in the seeds of the white grape variety Tsolikauri: gallic acid (0.535 mg/g), syringic acid (0.313 mg/g), and protocatechuic acid (0.212 mg/g) [52]. Individually, the most important hydroxybenzoic acids in grapes are gallic, vanillic, and syringic acids [77]. Gallic acid is considered the most important phenolic acid, being the precursor of all hydrolysable tannins [78]. The gallic acid content in the grape bunch was 5 times lower, while syringic acid was not detected [52]. Gallic acid was also detected in the whole grapes of the dark-pink variety Chkhaveri [74]. Stilbenes are formed via the condensation of three C2 carbon residues with an activated hydroxycinnamic acid [79]. These compounds consist of two aromatic rings linked by an ethylene bridge, forming the core diphenylethylene structure (C6-C2-C6) [79]. In grapes, stilbenes are mainly present in the skin, and red varieties are characterized by a higher content of stilbenes than the white ones [80]. The main grape stilbenes are *cis*- and *trans*-resveratrol (3,5,4′-trihydroxystilbene), resveratrol-3-*c*-β-d-glucopyranoside (piceid) [81]. Trans-resveratrol glucoside, trans-piceid (4′, 5-dihydroxylstilbene-3-*O*-β-D-glucopyranoside) was identified in 9 varieties of red grapes, and the concentration of this compound in grape juice was much higher than in grape skin (2.20 mg/L–12.41 mg/L versus 0.12 mg/kg–0.94 mg/kg) [58]. Resveratrol was detected in the skin and seeds of the red grape variety Saperavi, and its content in seeds was significantly higher than in skin (400 mg/kg).

### 2.3. The “Qvevri” Method of Wine Production and Its Implications for Wine Chemistry

**Figure 3:**
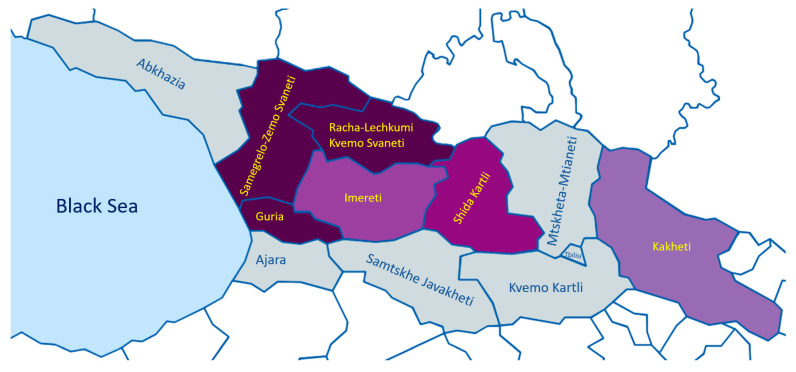
Map of winemaking regions of Georgia according to the National Wine Agency of Georgia [84]. Different colors represent the proportion of white and red winemaking grape varieties (darker shades of purple represent a higher proportion of red versus white grape varieties).Samegrelo Zemo Svaneti—1 Red winemaking grape variety; Guria—3 red winemaking grape varieties; Racha-Lechkumi Kvemo Svaneti—3 red winemaking grape varieties; Imereti—2 red and 3 white winemaking grape varieties; Shida Qartli—2 red and 2 white winemaking grape varieties; Kakheti—2 red and 4 white winemaking grape varieties.

The distinct diversity between wines of different Georgian regions is due not only to various natural and climatic conditions, but also to various grape varieties and specificities of wine technologies [82]. The regions of Georgia are distinguished into regions producing either mainly red or white wines or regions producing both wine types. More than 60% of viticultural production is concentrated in the Kakheti area [82], which is the most important Georgian winemaking region, known for the highest quality wines [83]. According to the National Wine Agency of Georgia [84], there are 13 red wine grape varieties and 9 white wine grape varieties across different regions of Georgia, making a total of 22 widely used winemaking grape varieties (Figure 3).

**Figure 4:**
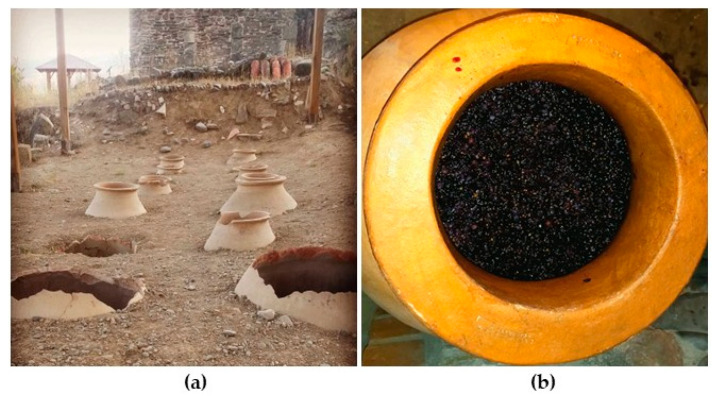
Photo of “qvevri” vessels. (**a**) Ancient “qvevri” vessels in Bagineti, Georgia (41.8374519721305, 44.721669015503); (**b**) Fermentation in “qvevri” in Kapito Cellar Zemo Khandaki, Georgia (41.905272341209674, 44.31120046133261).

The Georgian traditional “Kakhetian” winemaking method involves the fermentation of grape juice with the pomace (skins, seeds, peduncles and stalks), called “chacha”. The grapes are crushed using a traditional wooden wine press, “satsnakheli”, by stomping on them with the feet [85]. This wooden press is placed near the vessel for wine fermentation, and the juice flows into the vessel via a special gutter. The traditional Georgian vessel, used for winemaking, is called “qvevri” (Figure 4). It is a large clay vessel buried in the ground [86]. During the fermentation (10–20 days), the “qvevri” remains uncovered to allow immersion of the pomace cap [82]. Once fermentation is over and yeast activity ceases (this moment is indicated by the immersion of the pomace cap), the oxygen diffusion is limited by sealing with two layers: one of clay and another of sand, placed on top of a stone [87]. Afterwards, the maturation phase starts, which can range from one to several months and can take place either in the same “qvevri” or in another clean “qvevri” until the wine is ready for bottling [88].

Several wine styles are based on “qvevri”: (1) the “Kakhetian style”, where the fermentation of must is performed by adding up to 100% “chacha”; (2) the “Imeretian style”, where the fermentation takes place in “qvevri” with the partial (2.5–3.0%) addition of “chacha”; (3) “European style” without addition of “chacha”; (4) the “Naturally semi-sweet wines” as well as sparkling wines [86]. The “chacha” also undergoes a process known as punch down twice a day during fermentation [87]. This is crucial for extracting the desired compounds, such as phenols, and helps regulate the temperature to prevent overheating, which can cause yeast death. The places where “qvevri” are placed are called “marani”, and two types of “marani” cellars are known in Georgia: closed type (eastern Georgia) and open type (western Georgia). The closed “marani” is built of stone, and the “qvevri” vessels are buried in the soil up to the neck in this case. In the open “marani” cellar, “qvevri” vessels are also buried in soil, but in an open-air place [85]. The burying of “qvevri” vessels in the ground provides a consistent temperature regimen and stability, which helps regulate the fermentation process and the molecular composition of the resulting wine [89]. The ancient Georgian winemaking tradition based on the “qvevri” vessels, in accordance with the principles of the Convention on Protection promoted by UNESCO, received the status of National Monument of Intangible Cultural Heritage in 2013 [90]. From a molecular perspective, the “qvevri” winemaking has a direct connection to the chemical properties of wine, and these chemical properties also influence how other foods, treated with wine in the Georgian tradition, can be preserved and enriched when aged in wine. Wine made in “qvevri”, the traditional Georgian clay vessel, develops a different chemical composition compared to wine made in metal tanks or wooden barrels. In fact, as anticipated, the clay walls allow very slow oxygen movement, and the wine is usually fermented together with grape skins, seeds, and sometimes stems. Because of this, “qvevri” wines, especially red varieties like Saperavi, contain high levels of phenolic compounds, anthocyanins, tannins, minerals, organic acids, and antioxidants [91]. Famously, these compounds are important because they exhibit strong biological activity, with polyphenols and anthocyanins from red wine being known for their radical-scavenging and antimicrobial properties. In fact, they can neutralize free radicals, slow oxidation, and resist harmful microorganisms. Interestingly, when Georgian cheese is aged in Saperavi wine, many of these compounds migrate from the wine into the cheese, with specific experiments showing that the cheese rind, which remains in contact with the wine, absorbs the largest amount of phenols and anthocyanins [91]. Cheese that has been kneaded with wine before aging absorbs even more of these compounds. As a result, Georgian wine does not simply add flavor or color, it improves food’s stability and preservation as the antioxidants from Saperavi wine slow down the oxidation of fats in the cheese, and the natural acidity and phenolic content help reduce microbial spoilage. The combination of wine polyphenols and dairy proteins also creates a richer nutritional profile. Together, they provide more complete amino acids, more antioxidants, and substances that may have anti-inflammatory, anti-edematous, and even anticancer effects [91]. This means that wine-aged food is not only preserved more effectively but may also have increased dietary and functional value. Similarly, phenolic compounds can be transferred from Georgian wine to bread, contributing to the high biological activity of the resulting bread that can serve as a functional food with therapeutic and prophylactic applications in human diet [92].

### 2.4. Antioxidant Activity and Total Phenolic Content of Georgian Wines

**Figure 5:**
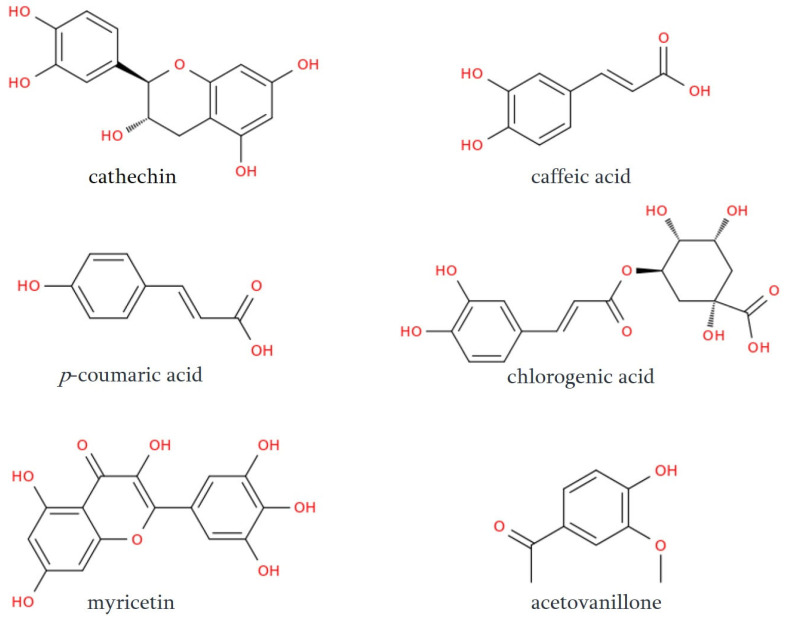
Chemical representations of selected phenolic compounds present in the Saperavi grapevine.

A growing body of analytical research demonstrates that the phenolic composition of red wine, as well as the concentration of individual components, plays a defining role in its physiological properties and sensory behavior. Recent comparative studies employing HPLC-UV analysis of Cabernet Sauvignon and Saperavi wines produced across multiple regions and vintages consistently highlight the prominence of several key polyphenols in Saperavi [93]. While both wines contain the same major classes of phenolic constituents, including hydroxycinnamic acids, flavones, flavan-3-ols and stilbenoids, the quantitative profiles reveal that Saperavi accumulates substantially higher amounts of several bioactive compounds. Meta-analytic synthesis of available datasets shows that catechin (Figure 5) levels in Saperavi often exceed those of Cabernet Sauvignon by approximately 25–40% (e.g., typical ranges of 120–150 mg/L in Saperavi vs. 80–110 mg/L in Cabernet Sauvignon), while caffeic acid frequently is 30–50% higher, reaching 8–12 mg/L in Saperavi compared to 5–8 mg/L in Cabernet Sauvignon. Likewise, *p*-coumaric acid displays an increase of roughly 20–35%, chlorogenic acid rises by 30–60%, and myricetin, one of the dominant flavonols, tends to be 40–70% more abundant in Saperavi, often reported near 12–18 mg/L compared to 7–11 mg/L in Cabernet Sauvignon [93].

Altogether, the assembled analytical evidence positions Saperavi as a phenolic-rich wine distinguished by significantly elevated concentrations of several structurally and biologically important compounds. These higher levels, together with the more coherent patterning of correlations among flavonols and stilbenoids, highlight the characteristic metabolic profile of the Saperavi grape and its notable capacity to accumulate abundant polyphenolic material [93]. Acetovanillone (Figure 5) is another example of polyphenol obtained from Saperavi grapevine. In particular, it was detected in the water–ethanol extract of Saperavi vine stems at a concentration of 5.2 mg/L. Due to its radical-scavenging action, and its anti-inflammatory, anti-rheumatic, and NADPH-oxidase-inhibiting properties, the presence of acetovanillone in the extract, and, therefore, in the biologically active supplement, contributes positively to its curative and prophylactic value [94]. Owing to the biological properties of Georgian wine, a comparative study of antioxidant activity of 26 red wines and 13 white wines commonly produced in Georgia and Central and Western Europe by DPPH radical-scavenging assay and oxygen radical absorbance capacity (ORAC) assay revealed the highest radical-scavenging activity in red wines from the Georgian variety Saperavi and cuvee Saperavi + Saperavi Budeshuriseburi (range of DPPH and ORAC were 0.59–2.08 g TE/L wine and 12.14–7.29 g TE/L wine) [7]. As it was shown previously, red wine exhibits the highest antioxidant activity, followed by rose and white wines [95]. The same pattern remained in the discussed study, except for two samples of Rkatsiteli white wine, obtained by fermentation in “qvevri” [7]. The highest antioxidant activity in these white wine samples was comparable to some red wines (DPPH, ORAC of 2.90 g TE/L wine and 4.01 g TE/L wine). The comparison of Georgian wines, obtained by fermentation in “qvevri” vessels and the classical European method, also revealed a drastic difference in radical-scavenging activities [10,11]. In general, the radical-scavenging activity was significantly higher in wines produced in “qvevri” vessels in comparison with wines obtained by the classical method: this difference was almost two-fold for Rkatsiteli, Kisi, and Khikhvi white wines, while the difference was not so drastic, but still statistically significant for Saperavi red wine [10,11]. The difference in the antioxidant activity determined by the FRAP method was 13-fold higher for Rkatsiteli produced by the “qvevri” method in comparison with Rkatsiteli produced by the European method, while for Saperavi wine “qvevri” method of production resulted in a 1.8-fold increase in comparison with the traditional method [96]. The high antioxidant activity correlated with high total phenolic content (TPC) in wines produced by the “qvevri” method (Table 2). Thus, TPC in red Saperavi wines obtained by the “qvevri” method on average was significantly higher than that of Saperavi produced by the classic European method (Table 2) [7,11,96]. For white wines, the difference between TPC of wines obtained by “qvevri” and European method was even more significant: it was at least 5-fold higher for Rkatsiteli produced by the “qvevri” method and 3–4 times higher for Kisi and Khikhvi wines obtained by the “qvevri” method (Table 2) [7,10,11,96].

Remarkably, white wines produced using Kakhetian technology contain abundant bioactive compounds and demonstrate stronger antioxidant activity and inhibitory effects on pancreatic lipase activity compared to those crafted with traditional European methods [92].

Since the use of lipase inhibitors to reduce dietary fat absorption and to develop anti-obesity agents is considered an attractive approach, and currently represents one of the main strategies in the management and treatment of obesity [97], these findings highlight the potential health relevance of Kakhetian wines.

## 3. The Phenolic Composition of Georgian Wines

The most common flavonoids in wine are flavan-3-ols, flavonols and in red wines, anthocyanins [98]. The most important property of almost any group of flavonoids is their ability to act as radical-scavengers [99] and the presence of the B-ring and-orthocatechol group determines this ability [100]. For flavonoids lacking a catechol group, the basic structure becomes important, i.e., the presence of both a 2,3-double bond and a 3-OH group is fundamentally important for a high antioxidant activity by facilitating electron delocalization and radical stabilization [101]. This conclusion was confirmed by the similar radical-scavenging activity of the quercetin (flavone) and catechin (flavan-3-ol) flavonoids, which possess different basic structures but the same hydroxylation pattern (3,5,7,3′,4′-OH) [100]. However, the flavone kaempferol (3,5,7,4′-OH), without a catechol group, also demonstrated a high antioxidant activity, attributed to the presence of both the 2,3-double bond and the 3-hydroxyl group [100]. Also, the position, structure, and total number of sugar moieties in flavonoids are important for the antiradical activity, with aglycones being more potent antioxidants than their corresponding glycosides [102]. In the subclass of flavan-3-ols, the primary monomeric units in grapes and wine are catechin and epicatechin; however, various derivatives, such as epicatechin gallate and epigallocatechin gallate, were revealed in both monomeric and polymeric forms [103]. Epigallocatechin gallate and catechin were identified in Georgian wines produced by both the European and “qvevri” method (Table 3), and the content of catechin was significantly higher in red and white wines produced by the “qvevri” method [7,54,96,104]. Catechins possess high antioxidant activity due to the presence of a hydroxyl group, providing electron-donating capacity, enabling them to scavenge free radicals or chelate metal ions [105]. Therefore, catechins present significant potential as a therapeutic approach for cardiovascular diseases, and recent clinical studies demonstrated the beneficial effects of catechins in atherosclerosis, hypertension, heart failure and cardiomyopathy (reviewed in [105]), as well as type II diabetes [106]. Flavonols are exclusively present in grapes and wines as glycosylated forms (3-*O*-glucosides, 3-*O*-galactosides, and 3-*O*-glucuronides); free aglycones can be found together with the glycosylated forms [107]. Free aglycones are released from the glycosylated forms by hydrolysis during winemaking, and the degree of hydrolysis is dependent on the flavonols’ structure and the nature of the 3-*O*-glycoside [99]. Thus, the conditions of wine production can significantly change the flavonol profile of grapes. Quercetin and its conjugated derivatives are frequently the main flavonols in both red and white grapes [99]. The presence of quercetin glucuronide was shown in some white European wines; however, no quercetin, isoquercitrin, rutin, or kaempferol-3-*O*-glucoside was detected in any of the wines analyzed [108]. Interestingly, in Georgian white wines, flavonols were not detected [7,54,104,109] (Table 3). A comparative study of European wines revealed the high abundance of quercetin, rutin, and myricetin. The presence of quercetin glucuronide, quercetin glucoside and kaempferol glucoside was also shown (reviewed in [108]). In Georgian red wines produced by both the European and the “qvevri” method, quercetin glucuronide, quercetin glucoside, kaempferol rutin, and myricetin were detected using HPLC-UV/Vis method (Table 3). Kaempferol was the most abundant flavanol in Saperavi red wine produced by the “qvevri” method, while myricetin was the most abundant flavanol in Saperavi and Kindzmarauli red wines produced by the European method [7,54,104,109]. As for the resulting biological properties of these compounds, kaempferol was shown to be responsible for estrogenic activity in red wine, demonstrating the highest affinity for estrogen receptors, as was demonstrated using ligand binding and yeast transactivation assays [110] and possesses antimicrobial properties against various strains of Gram-positive and Gram-negative bacteria in vitro [111]. On the other hand, myricetin exhibits anticancer activity as was demonstrated in studies of compound effect on hepatic, skin, pancreatic, and colon cancer cell lines, antidiabetic and anti-inflammatory activities [112]. The antidiabetic activity of myricetin was due to its inhibitory action on the aggregation of islet amyloid polypeptide, known to play a major role in the death of pancreatic β-islet cells in type II diabetes [112]. The anti-inflammatory activity of myricetin has been demonstrated in multiple in vitro assays, as well as animal models of both acute and chronic inflammation [113].

Flavones and flavanones are rarely reported in wines, with the only two detected flavones in wines being apigenin and luteolin [115]. In Georgian white wines, flavones were not detected, while luteolin was revealed in red wines produced by both the European and the “qvevri” method. Moreover, apigenin was detected only in one wine sample, produced by the European method (Table 3) [54]. Both luteolin and apigenin possess radical-scavenging properties in vitro; however, their antioxidant mechanism has not been fully revealed [116]. Anti-inflammatory and neuroprotective properties were demonstrated for luteolin in studies on cell lines and mouse models [117]. Famously, anthocyanins are the main phenolic compounds involved in the color of red wines [118]. In the red wines, the main monomeric anthocyanins are the 3-*O*-monoglucosides of the six free anthocyanidins, including pelargonidin-3-*O*-glucoside, cyanidin-3-*O*-glucoside, delphinidin-3-*O*-glucoside, peonidin-3-*O*-glucoside, petunidin-3-*O*-glucoside and malvidin-3-*O*-glucoside [119]. These anthocyanidins differ by the number and positions of the hydroxyl and methoxyl substituent groups on the B ring. The hydroxylation pattern of the anthocyanins in the B ring affects the color stability: anthocyanins with more hydroxyl groups in the B rings contribute more blueness, and the degree of methylation of the B rings can increase the redness, with these substitution patterns further regulating antiradical activity, and influencing copigmentation interactions, as well as pH-dependent wine color expression [119]. Thus, the malvidin-3-*O*-glucoside and its derivatives are the reddest anthocyanins [120], mainly determining the wine’s color. The analysis of red Georgian wines by HPLC-UV/Vis method indicated that in the majority of wines obtained by the “qvevri” method, the most abundant anthocyanins in Sapervai, Alexandouli, Mujuretuli, Ojaleshi, and Otskhanuli Sapere red wines were delphinidin-3-*O*-glucoside, peonidin-3-*O*-glucoside, petunidin-3-*O*-glucoside and malvidin-3-*O*-glucoside [109,121], while a high content of cyanidin-3-O-glucoside was revealed only in Ojaleshi red wine sample [121], (Table 4). The main anthocyanin detected in all analyzed wines was malvidin-3-*O*-glucoside. Importantly, the interaction between this glucoside and flavan-3-ols is responsible for the color and flavor of red wine [122]. It also possesses strong antioxidant properties, and its protective effects against peroxynitrite-promoted apoptotic death of endothelial cells and DNA damage induced by chemotherapeutic drugs in vitro were also demonstrated [123].

The content of non-flavonoids in wine usually increases with increased time of maceration [124]. Therefore, wines prepared by the traditional “qvevri” method are usually characterized by a higher content of these compounds. Thus, vanillic acid content in South American wines varied from 0 to 1.15 mg/L, in Georgian wines it comprised 1.89 to 8.32 mg/L. For South American wines, caffeic acid content was 2.74–4.95 mg/L, and in Georgian red wines it was 6.89 to 80.55 mg/L. Trans-resveratrol content was 1.56–4.30 mg/L in South American wines versus 7.92–18.65 mg/L for Kindzmarauli and Saperavi [54].

Phenolic acid content in wines influences not only the flavor balance, but also factors such as the chemical stability and pH, and consequently, the quality of the wine [125]. Hydroxycinnamic acids in wines exist in two isomeric forms (*cis* and *trans*). Hydroxybenzoic and hydroxycinnamic acids not only occur in their free forms but are also present as derivatives in conjugated or esterified forms [124]. For example, hydroxycinnamic acids in wine originate during fermentation from the hydrolysis of hydroxycinnamic tartaric esters [126]. The comparison of wines prepared from Georgian varieties using the European method and the traditional “qvevri” method revealed that in white wines, prepared by both methods, the most abundant hydroxybenzoic and hydroxycinnamic acids were gallic and caffeic acids; in red wines, the most abundant were gallic and chlorogenic acids (wines, prepared by both methods), and syringic and caftaric acids for red wines, prepared by “qvevri” method (Table 5) [7,54,96]. Earlier, caftaric acid was shown to be the predominant phenolic acid in European white wines, while caffeic and gallic acids were considered important hydroxycinnamic and hydroxybenzoic acids [127]. Interestingly, in Georgian white wines, produced by both European and “qvevri” method, caftaric acid was not detected [7,96]. Gallic acid was earlier reported as the main hydroxybenzoic acid in red wines, possessing antimicrobial, antioxidant, anticancer, anti-inflammatory, and antiviral properties, which was demonstrated in numerous in vitro studies [128]. Caffeic acid was extensively studied in white wines and numerous in vitro and animal models studies demonstrated its protective effect against a wide range of toxins, including mycotoxins, heavy metals, pesticides, industrial chemicals, and pharmaceuticals [129]. Chlorogenic acid is always present in wine [130], causes modulation of anti-inflammation/oxidation and metabolic homeostasis, thus possessing neuroprotective action for neurodegenerative disorders and diabetic peripheral neuropathy, anti-inflammatory, anti-oxidative activities demonstrated in human subject studies, anti-pathogenic, and anti-tumor activities demonstrated in vitro [131].

**Scheme 1:**
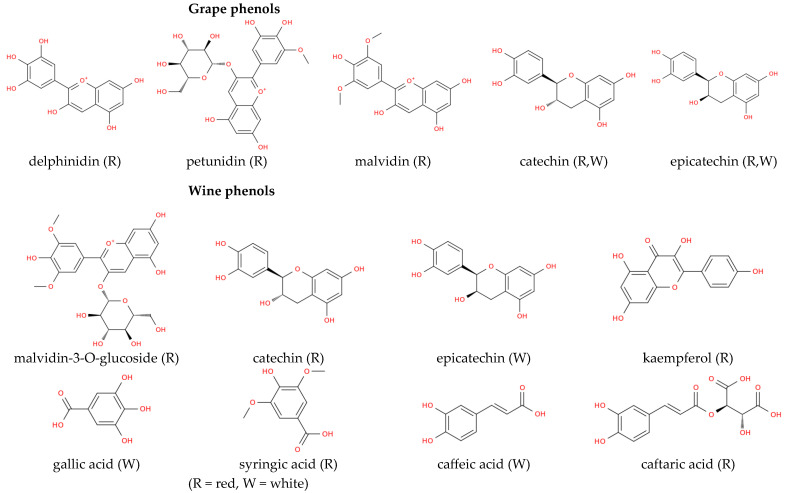
Chemical structures of the principal phenolic compounds occurring in grapes and wines, with a specific focus on those most characteristic of red and white wines obtained through “qvevri” vinification.

It is worth noting that stilbens were revealed in both grapes and wines. In this respect, resveratrol is the primary stilbene in grapes, and the *trans*-isoform predominates, while in wine, both *cis*- and *trans*-resveratrol are present [132]. In all Georgian red wines produced by both the European and the “qvevri” method, only *trans*-resveratrol was revealed, while other stilbenes were not detected in white wines (Table 5), [109]. Higher concentrations of resveratrol in red wines compared with those produced from white varieties were demonstrated [133]. Remarkably, as demonstrated by in vivo and vitro studies, resveratrol has antioxidant, anti-inflammatory, anti-cancer, immunomodulatory, glucose and lipid regulatory, neuroprotective, and cardiovascular protective effects [134]. Overall, the phenolic composition of Georgian wines has a key role in their potential utility as functional beverages as suggested by the biomedical applications demonstrated for their components. Not least importantly, these findings reinforce the value of plant-derived natural products, including compounds from plants and other sources, as promising candidates for biomedical research and therapeutic development against a range of diseases [134]. The most abundant phenolic compounds of red and white Georgian grape varieties and red and white Georgian wines are represented in Scheme 1.

The significant variations in the content of phenolic compounds presented in this review are due to a combination of grape variety, vineyard environmental factors (terroir), and winemaking techniques. As was discussed at the beginning of the review, grape variety, grape maturity, as well as climate and geographic origin have a significant impact on the content of phenolic compounds. Winemaking techniques such as maceration, fermentation temperature control, and oak aging were also recognized as key factors of such variations [135]. The results discussed in the review demonstrate that the qvevri method, characterized by prolonged maceration, where grape solids (skins, seeds, stems) stay in contact with the juice for weeks or months, enhances the extraction of phenolic compounds. The clay qvevri vessels provide thermal stability and consistent low temperatures, which aid extraction. In addition, the traditional qvevri method, involving punch-downs of the pomace cap, mechanically breaks up solids to maximize phenolic release.

## 4. Conclusions

Both red and white Georgian grape varieties contain a broad spectrum of phenolic compounds, mainly in the seeds, skin, and stalk. The comparison of wines produced by European and “qvevri” methods from Georgian grape varieties revealed that the higher radical-scavenging activity of both white and red wines produced by the “qvevri” method correlates with higher total phenolic content. From a molecular perspective, both white and red wines prepared by the “qvevri” method were characterized by a higher content of catechin. In red wines prepared by the “qvevri” method, higher contents of kaempferol and malvidin-3-*O*-glucoside were also revealed. These representatives of various classes of flavonoids exhibit significant antioxidant activity, providing various health protective effects. Hence, the obtained results of this literature review suggest that the antioxidant effect of Georgian wines is determined by the content and composition of phenolic compounds that, in turn, depend on the traditional winemaking procedure. In fact, the composition of phenolic components in wine is determined not only by the grape variety but also shows a close correlation with the winemaking technology, as highlighted in this work. Although alcohol is recognized as a biological toxin and a substantial body of literature discourages the consumption of alcoholic beverages [136], the moderate, daily use of wine is frequently documented among semi-centenarian and centenarian individuals [137] and can be interpreted not only as a source of antioxidant and potentially anticancer compounds [138], but also as a contributor to social bonding and community interaction in regions with long-standing wine-drinking traditions, such as Georgia. Interestingly, this study emphasizes the potential of Georgian wines as functional beverages with nutraceutical value and highlights the need for further research on the phenolic composition of unexplored Georgian grape varieties, as well as on methodological variations in the traditional “qvevri” winemaking process, intending to produce wines endowed with maximal radical-scavenging activity and health-promoting effects.

## Figures and Tables

**Table 1:** Distribution of phenolic compounds in different grape parts of Georgian white (W) and red (R) grape varieties.

Class/Subclass of Phenolic Compounds	Variety	Skin	Seeds	Stalk	Ref.
**Flavonoids** ***Flavan-3-ols***	Tsolikauri (W)	Catechin, epicatechin	Catechin, epicatechin	Catechin, epicatechin	[40]
	Rkatsiteli (W)	gallocatechin, epicatechin, catechin, epicatechin gallate	Epicatechin, catechin, epicatechin gallate	Gallocatechin, epicatechin, catechin, epicatechin gallate	[53]
	Saperavi (R)	catechin hydrate	Catechin hydrate		[54]
***Flavonols***	Tsolikauri (W)	Rutin	Rutin	Quercetin, rutin	[40]
***Anthocyanins***	Saperavi (R)	Delphinidin, petunidin, malvidin, peonidin, delphinidin-3-*O*-glucoside			[55,56]
**Non-flavonoids** ***Phenolic acids***	Tsolikauri (W)		*Hydroxycinnamic acids*: ferulic acid; *Hydroxybenzoic acids*: gallic, protocatechuic, syringic acids	*Hydroxycinnamic acids*: caffeic acid; *Hydroxybenzoic acids*: gallic acid	[57]
***Stilbenes***	Saperavi (R), Cabernet Sauvignon (R), Otskhanuri Sapere (R), Aleksandrouli (R), Mujuretuli (R), Shavkapito (R), Tavkveri (R), Aladasturi (R), Dzelshavi (R), Ojaleshi (R)	4′, 5-dihydroxylstilbene-3-*O*-β-D-glucopyranoside, resveratrol	Resveratrol		[54,58]

**Table 2:** TPC, expressed as grams of Gallic Acid Equivalent (GAE) per liter, in Georgian red (R) and white (W) wines from different methods.

Wine	Production Method	TPC, g L^−1^	Reference
**Saperavi (R)**	European method	1.1–4.5	[7,11]
	“qvevri” method	2.7–4.4	[7,11,96]
**Rkatsiteli (W)**	European method	0.2–0.5	[7]
	“qvevri” method	2.0–2.5	[11]
**Kisi (W)**	European method	0.7	[11]
	“qvevri” method	2.2	[11]
**Khikhvi (W)**	European method	0.4	[11]
	“qvevri” method	1.6	[11]

**Table 3:** The content of various subclasses of flavonoids (mg/L) in Georgian red (R) and white (W) wine, produced by European (E) and “qvevri” (Q) methods, determined by HPLC-UV/Vis.

Content of Flavan-3-ols, mg/L	Rkatsiteli (W, E)	Rkatsiteli (W, Q)	Saperavi (R, E)	Saperavi (R, Q)	Kindzmarauli (R, E)	Ref.
*Epicatechin*	17.8–29.0	38.7–58.6	9.0–76.2	29.5–62.2	118.5	[54,96,104]
*Epigallocatechin gallate*	1.4–2.0	1.7–22.9	15.0–56.3			[7]
*Gallocatechin*		43.7		174.4		[104]
*Catechin*	17.8–29.0	32.6–45.2	5.0–272.1	5.0–387.8	221.1	[54,96,104]
**Content of flavanols, mg/L**						
*Quercetin glucuronide*				9.7		[109]
*Quercetin glucoside*			9.8–14.3	4.3–12.3	7.1	[54,109]
*Myricetin*	0	0	0–69.4	0	63.5	[7,54]
*Kaempferol*	0	0	0.4–2.5	3.2–13.2	0.8	[7,54,104]
*Rutin*	0	0	0–4.1	2.6		[7]
**Content of flavones, mg/L**						
*Apigenin*			0–1.06	0	0	[54]
*Luteolin*			0.56–0.96	0.75	1.15	[54]
**Content of stilbenes, mg/L**						
*Trans-resveratrol*			10.1–18.7	11.6–13.8	7.9	[54,114]
*Trans-resveratrol glucoside*				69.0		[109]
*Cis-resveratrol glucoside*				13.8		[109]

**Table 4:** The content of anthocyanins (mg/L) in Georgian red wines produced by “qvevri” methods, determined by HPLC-UV/Vis.

Content of Anthocyanins, mg/L	Wines					
	Saperavi	Alexandrouli	Mujuretuli	Ojaleshi	Otskhanuli Sapere	Reference
*Delphinidin-3-O-glucoside*	47.8–72.0	11.2–52.1	13.6	41.3	34.6	[109,121]
*Cyanidin-3-O-glucoside*	1.5–6.9	6.6–11.7	5.4	89.4	34.6	[109,121]
*Petunidin-3-O-glucoside*	71.0–92.4	92.4	39.3	77.3	53.2	[109,121]
*Peonidin-3-O-glucoside*	27.7–92.0	27.5–47.7	21.9	46.3	12.9	[109,121]
*Peonidin-3-O-acetylglucoside*	16.0–33.1	7.0–15.8	13.7	19.7	4.7	[109,121]
*Peonidin-3-O-p-coumarylglucoside*	34.1	34.4–94.6	25.0	9.4	14.6	[121]
*Malvidin-3-O-glucoside*	264.1–340.0	171.5–353.1	125.4	126.9	136.8	[109,121]
*Malvidin-3-O-coumarylglucosid*	9.4–44.0	7.4–19.9	4.9	2.5	3.25	[109,121]

**Table 5:** The content of various subclasses of non-flavonoids (mg/L) in Georgian red (R) and white (W) wines produced by European (E) and “qvevri” (Q) methods, determined by HPLC-UV/Vis.

Content, mg/L	Rkatsiteli (W, E)	Rkatsiteli (W, Q)	Saperavi (R, E)	Saperavi (R, Q)	Kindzmarauli (R, E)	Reference
**Hydroxybenzoic acids**						
*Gallic acid*	7.1–581.3	9.9–136.0	5.0–101.4	4.8–21.8		[7,54,96]
*Syringic acid*	0.2–0.5	0.2–0.3	4.7–12.6	22.00		[7,109]
*Vanillic acid*	0.2–0.6	0.4–0.5	1.9–9.1	8.3	5.86	[7,54]
*3,4-hydoxybenzoic acid*	0.8–2.7	1.3–3.2	1.9–9.1			[7]
*Protocatechuic acid*						[104]
**Hydroxycinnamic acids**					14.4	
*Caffeic acid*	5.3–91.6	0.9–111.7	3.2–108.3	7.4–80.5	55.6	[7,54,104,109]
*p-coumaric acid*	1.0–3.3	0.7–0.8	2.8–16.5			[7,54]
*Chlorogenic acid*	2.5–2.8	2.0–3.2	1.8–109.9	138.8		[7]
*Ferulic acid*	0.3–0.6	0.2	0.3–1.8			[7]
*Caftaric acid*				158.0		[109]
*Courtaric acid*				117.0		[109]
**Stilbens**						
*Trans*-resveratrol			10.1–18.7	11.6–13.8	7.9	[54,114]
*Trans*-resveratrol glucoside				69.0		[109]
*Cis*-resveratrol glucoside				13.8		[109]

## Data Availability

The original contributions presented in this study are included in the article. Further inquiries can be directed to the corresponding authors.
